# Polydioxanone Membrane Compared with Collagen Membrane for Bone Regeneration

**DOI:** 10.3390/polym15040868

**Published:** 2023-02-09

**Authors:** Lilian Caldas Quirino, Pedro Henrique de Azambuja Carvalho, Renato Torres Augusto Neto, Cássio Amaro Comachio, Naara Gabriela Monteiro, Ana Cláudia Ervolino-Silva, Roberta Okamoto, Valfrido Antonio Pereira-Filho

**Affiliations:** 1 Department of Diagnosis and Surgery, School of Dentistry, São Paulo State University (UNESP), Rua Humaitá 1680, Araraquara CEP 14801-903, SP, Brazil; 2 Department of Diagnosis and Surgery, School of Dentistry, São Paulo State University (UNESP), Rua José Bonifácio, 1193, Araçatuba CEP 16015-050, SP, Brazil; 3 Department of Basic Sciences, School of Dentistry, São Paulo State University (UNESP), Rua José Bonifácio, 1193, Araçatuba CEP 16015-050, SP, Brazil

**Keywords:** polydioxanone, bone regeneration, membrane, synthetic polymer

## Abstract

Guided bone regeneration (GBR) is an approach that induces osteopromotion through the regenerative membranes. These barriers exhibit bioactive behavior and mechanical function. Polydioxanone is a synthetic option, already used in medicine and dentistry, with good results in bone regeneration. This study aimed to evaluate bone repair in critical defects in rat calvaria using a polydioxanone membrane (Plenum^®^ Guide) compared with a commercially available collagen-based membrane (Bio-Gide^®^). The bone defects were filled with Plenum^®^ Oss*hp*, a synthetic bone graft, hydroxyapatite:β-tricalcium phosphate, 70:30%, Group PG (Plenum^®^ Guide + Plenum^®^ Oss*hp*), and Group BG (Geistlich Bio-Gide^®^ + Plenum^®^ Oss*hp*). The specimens were submitted to immunohistochemical (RUNX2 and OPN), gene expression (RUNX2, IBSP, and VEGF), histometric, and microtomography analyses after 07, 15, 30, and 60 days postoperative. PG group showed greater immunolabeling area for RUNX2 and OPN, higher gene expression of VEGF (3.15 ± 0.85), and IBSP (24.9 ± 0.59). However, there was no statistical difference between groups in the histometric analysis regarding the percentage of connective tissue PG (0.83 ± 0.45), BG (0.70 ± 0.34), neoformed bone PG (0.60 ± 0.4), BG (0.65 ± 0.51), and remaining biomaterial PG (0.84 ± 0.31), BG (0.91 ± 0.33). In addition, there was no statistical difference between groups by micro-CT analysis. The absorbable-synthetic membrane, Plenum^®^ Guide, is an effective membrane for guided bone regeneration.

## 1. Introduction

In the early 1980s, guided tissue regeneration (GTR) therapy was established to redirect fibroblasts from the periodontal ligament and promote periodontal regeneration in chronic periodontitis situations [1]. Currently, the GTR technique has been explored frequently in periodontal surgery, implantology, and craniomaxillofacial reconstructions. 

The success of oral rehabilitation using osseointegrated implants is directly related to the quantity and quality of bone tissue at the implant site. Unfortunately, the daily dental practice commonly finds alveolar bone defects caused by prolonged dental absence, periodontitis, tumors, or trauma. These defects can affect the longevity of implants as well as the aesthetics and function of rehabilitation [2]. In this situation, the technique of bone tissue regeneration is fundamental to achieve osseointegration, namely guided bone regeneration (GBR), which modulates the promotion of bone neoformation in bone defects through osteogenesis, osteoconduction, or osteoinduction, restoring the structural and functional characteristics of bone tissue [3]. 

The GBR associated with the implant placement procedure has shown high clinical predictability and effective gain in bone width and height, stabilizing implants and preventing fibrous tissue from adhering to their surface [4,5].

The therapeutic approach for GTR involves using different types of membranes (resorbable and non-resorbable) associated or not with various synthetic bone substitutes or grafts to fill or reconstruct bone defects [5,6,7,8]. Thus, the choice of biomaterials for GTR will depend on the size and conformation of the bone defect to be reconstructed, as well as the surgeon’s experience, previous knowledge, and skill in the surgical technique and handling of the materials [5]. 

The membranes, to be effective, must meet specific criteria, such as biocompatibility, integration with adjacent tissues, cell occlusion, mechanical and chemical stability, and easy usability [7]. Once the materials meet these critical factors of a membrane for GBR or craniomaxillofacial reconstructions with selective barrier function, the clinical results for tissue regeneration will be obtained safely and predictably.

Among the absorbable membranes, collagen-based membranes are the most commonly used for GBR due to their greater commercial availability and biocompatibility properties, besides promoting results with clinical predictability [7,9]. However, alloplastic materials stand out compared to materials of homogeneous or heterogeneous origin mainly due to the absence of risk of transmission of infectious diseases, absence of immunogenic responses, levels of religious beliefs [10,11,12], and more excellent uniformity of production compared to natural polymers (e.g., collagen, cellulose, alginate, chitosan, etc.); the by-products of degradation are acid molecules, which generally do not induce immune responses because they are typical by-products of cellular metabolism. Thus, synthetic absorbable polymers based mainly on aliphatic polyesters (PLA, PGA, and PLGA) or poly(ester-ether) (PDO) are commonly used in surgical procedures in the form of suture thread, rigid internal fixation systems (plates and screws), and as membranes for bone regeneration/repair [13,14]. The degradation of these materials occurs by hydrolysis and not by enzymatic route because the ester bonds are hydrolytically labile, and their hydrolysis occurs through a specific scission of these ester bonds. The by-products released from the reaction are natural and non-toxic metabolites (PLA, PGA, and PLGA: lactic and glycolic acids; PDO: glyoxylic acid or glyoxylate at neutral pH). The by-product of PLA is lactic acid, which is converted to pyruvate. The oxidative decarboxylation of pyruvate generates CO_2_ and acetyl-CoA (acetyl-CoA acts as fuel for the citric acid cycle in cellular respiration), so the by-products of PLA are converted to CO_2_ and water. The derivatives, glycolic acid from PGA or PLGA and glyoxylate from PDO, can either be eliminated directly in urine or converted to glycine/serine and, subsequently, converted to water and CO_2_ [13,15,16]. Polymer degradation factors (α-esters) depend on the product’s porosity, crystallinity, molecular weight, and surface area. The PLGA copolymer is preferred over its constituent homopolymers (PLA and PGA) for the manufacture of bone substitutes, as PLGA offers superior control of degradation properties by changing the ratio of LA and GA monomers. The various degradation rate is characteristic of PLGA and is directly related to polymer chain composition, hydrophobic/hydrophilic balance, and crystallinity [13,14,17,18]. Although PLGA membranes have provided good results, it allows control of degradation time by manipulating the proportions of lactic acid and glycolic acid, in addition to allowing the addition of bioactive factors, such as stem cells, vascular endothelial growth factor (VEGF), platelet-rich-plasma (PRP), and VEGF, enhancing bone repair [19]. On the other hand, the disadvantages of these polymers are the accumulation of acid released from the degradation by-product adjacent to the host tissue, which may generate undesirable inflammatory responses due to the acidic pH at this material–tissue interface [16,17]. In addition, they may have a lower degradation rate compared to PDO [15,16] due to the lower amount of ester groups. PDO polymer is a polymer synthesized by the precursor monomer p-dioxanone (multiple units repeated ether-ester polymer), which is semi-crystalline (crystallinity around 55%), and the ether group is responsible for the flexibility of the polymer chain [15,16,17,18,20], concerning polyesters. The PDO polymer can be resorbed entirely in 6 to 12 months, and the degradation by-products induce minimal or no foreign body reaction adjacent to the implant [21,22,23,24,25,26,27,28]. 

Plenum^®^ Guide is a polydioxanone-based absorbable synthetic membrane that presents a high potential in regenerative medicine, influencing the migration and adhesion of cells participating in the bone repair process. Given the advances in the field of tissue engineering, and the need to search for new biomaterials that are more efficient and affordable, the present study evaluated the performance of Plenum^®^ Guide on the bone repair process in critical calvaria defects compared with a collagen membrane.

## 2. Materials and Methods

### 2.1. Experimental Design

The model of bone repair of critical defects in rat calvaria was used to evaluate bone formation. Seventy-four critical defects were divided into two groups: 1. PG Group, in which the Guided Bone Regeneration (GBR) was performed using Plenum^®^ Guide (M3 Health Ind. Com. de Prod. Med., Odont. e Correlatos SA, Jundiaí, SP, Brazil) associated with Plenum^®^ Oss*_hp_* bone graft (synthetic–biphasic ceramic, hydroxyapatite:β-tricalcium phosphate, 70:30%, M3 Health Ind. Com. de Prod. Med., Odont. e Correlatos SA, Jundiaí, SP Brazil); 2. BG Group, in which the GBR was performed using Bio Gide^®^ (Geistlich Pharma, Wolhusen, Switzerland) associated with Plenum^®^ Oss*_hp_*.

At 7, 15, 30, and 60 days postoperatively, euthanasia was performed to evaluate calvaria bone repair by immunolabeling of RUNX2 (Runt-Related Transcription Factor 2) and OPN (Osteopontin) antibodies, relative gene expression of RUNX2, IBSP (Integrin Biding Sialoprotein) and VEGF (Vascular endothelial growth factor) proteins, histometric analysis of the percentage of new bone formed, connective tissue, remaining bio-material, and computed microtomography.

### 2.2. Animals and Ethics Committee

After approval by the Ethics Committee on Animal Use (CEUA43/2017), thirty-seven adult male rats (Rattus novergicus, Wistar) were randomly distributed into two experimental groups according to the barrier membrane used for the GBR, one corresponding to the PG group and the other to the BG group. Two surgical defects were created in the calvaria in each animal, totaling seventy-four evaluation sites.

The animals were kept in preoperative fasting for 8 h before the surgical procedure. Before any surgical procedure, the animals were anesthetized with ketamine hydrochloride (80 mg/kg, Fort Dodge, Animal Health, Ltda., Campinas, SP, Brazil) and xylazine (10 mg/kg, Coopers, Ltda., Osasco, SP, Brazil). Then, trichotomy and antisepsis of the calvaria region were performed. Local anesthesia was performed with lidocaine, without vasoconstrictor (20mg/mL, Cristália Produtos Químicos Farmacêuticos Ltda.; Itapira, SP, Brazil) diluted at 0.5% in saline solution at a 1-3 mg/kg dose.

**Figure 1:**
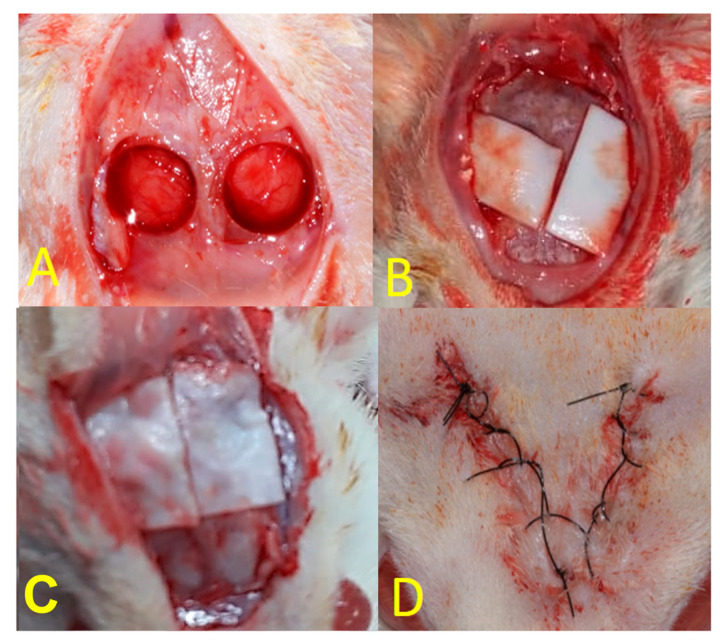
(**A**): Creation of critical defects in rat calvaria using a trephine with an internal diameter of 5 mm; (**B**): PG group; (**C**): BG group; (**D**): final aspect of the suture.

For each animal, a U-shaped incision was made in the occipitofrontal direction, and then, the flap was detached. Using a drill with an internal diameter of 5 mm (3i Implant Innovations Inc.; Palm Beach Gardens, FL, USA), two surgical defects were made in each calvaria, one in the left parietal bone and the other in the right parietal bone, maintaining the integrity of the dura mater (Figure 1A). Both groups filled the defects with Plenum^®^ Oss*_hp_* bone graft followed by the barrier membrane application. For the animals in the PG group, Plenum^®^ Guide was customized and applied over the filled defect (Figure 1B). For the BG group, the Bio Gide^®^ membrane was customized and used the same way over the filled defect (Figure 1C). The final dimensions of each membrane were 10 × 10 mm. Finally, the soft tissues were carefully repositioned and sutured (Figure 1D).

In the immediate postoperative period, each animal received a single intramuscular dose of 0.2 mL of benzathine penicillin G (Small Veterinary Pentabiotic; Fort Dodge Saúde Animal Ltda.; São Paulo, Brazil).

The animals were euthanized at 7, 15, 30, and 60 days after surgery using an overdose of anesthesia (sodium thiopental—100 mg/kg intraperitoneal dose).

### 2.3. Collection and Histological Processing

After euthanasia, the calvarias were collected and fixed in a 10% formaldehyde solution for 48 h, washed in running water for 24 h, and decalcified in 10% ethylenediaminetetraacetic acid (EDTA) and 20% sodium hydroxide, replaced every seven days for about 60 days. Samples were dehydrated in a series of ethanol solutions with increasing concentrations (70°–80° GL, 90°–95° GL, 100°–100° GL). Next, they underwent the process of dehydration in xylene and, finally, were infiltrated and embedded in paraffin. Histological sections (5 μm thick) were obtained in the sagittal plane.

Histological sections were stained by hematoxylin and eosin (HE) for histometric analysis of neoformed bone tissue, connective tissue, and biomaterial remaining from the critical defect site. Additional histological sections were subjected to indirect immunoperoxidase to detect RUNX2 and OPN.

Histological sections were deparaffinized in an oven for 20 min, followed by citrisol baths and baths of decreasing concentrations of alcohols, and then hydrated in PBS (phosphate-buffered saline 0.01 M). Endogenous peroxidase activity was inhibited with hydrogen peroxide. The slides were then subjected to antigenic recovery with citrate phosphate buffer (pH 6.0) in moist heat. Endogenous biotin was blocked with skim milk for 20 min. As a method to block nonspecific staining, the primary antibody was prepared in a 1% phosphate buffer in a solution of bovine albumin.

The primary antibodies used were polyclonal antibodies produced in goats against RUNX2 (SC8566 Santa Cruz Biotechnology, Santa Cruz, CA, USA) and OPN (SC 10593, Santa Cruz Biotechnology, Santa Cruz, CA, USA). The secondary antibody used was a biotinylated anti-goat antibody produced in donkeys (Jackson Immunoresearch Laboratories, West Grove, PA, USA). The signal of the reaction was amplified by incubation in avidin and biotin (ABC standard kit, Vector Laboratories, Burlingame, CA, USA), and the response was revealed using diaminobenzidine (Dako Laboratories, Santa Clara, CA, USA). A counterstain with Harris hematoxylin was performed at the end of the immunohistochemical reaction. Finally, histological sections were dehydrated in ethanol, clarified in xylene, and cover-slipped with per mount mounting medium (Permount, Fisher Scientific, San Diego, CA, USA) and glass coverslips for further analysis under an optical microscope (Nikon, Eclipse 80i, Shinagawa, Tokyo, Japan) with a 25x objective.

### 2.4. Micro-Computed Tomography (micro-CT)

The rat calvarias were removed, reduced, and fixed in a 10% formaldehyde solution for 48 h, washed in running water for 24 h, and then kept in a 70% alcohol solution. The samples were scanned using a SkyScan microtomograph (SkyScan 1272, Bruker MicroCT, Kontich, Belgium, 2003) in 13 μm slices with a 0.5 mm Al filter and a 0.6 mm rotation step. The scanning was performed at 180° with a frame of 2, a resolution of 2016 × 1344, and an acquisition time of approximately 50 min. The images, obtained by X-ray, were stored and reconstructed by determining the area of interest with NRecon software (SkyScan, version 1.6.6.0, 2011) using a smoothing of 2, an artifact ring correction of 5, a beam-hardening correction of 20%, and an image conversion range varying from 0.007 to 0.061. The images were reconstructed in Data Viewer (SkyScan, version 1.4.4, 64-bit) and observed on three planes (transversal, longitudinal, and sagittal). Then, using CT analyzer software (CTAn, 2003–11 and SkyScan, 2012; Bruker MicroCT; version 1.12.4.0), the region of the created defect (40 slices for each sample) was evaluated, using a histogram of 110–205 to remove the denser material. The parameters assessed were bone volume percentage (BV/TV), trabecular bone thickness (Tb. Th), the separation and the number of trabeculae (Tb. Sp and Tb. N), and connective density (Conn.Den).

### 2.5. Real-Time PCR

PCR was performed to evaluate the gene expression of markers related to the bone repair process of calvaria defects. PCR plates pre-designed by the manufacturer (Applied Biosystems, Foster City, CA, USA) were used to express tissue repair-related genes in bone formation and bone mineralization responses (Table 1).

Each bone fragment that contained repairing peri-implant bone was carefully washed in phosphate-buffered saline and frozen in liquid nitrogen to extract the total RNA using a Trizol reagent (Life Technologies: Invitrogen, Carlsbad, CA, USA). After an RNA integrity, purity, and concentration analysis, cDNA was created using one µg RNA via a reverse transcriptase reaction (M-MLV reverse transcriptase: Promega Corporation, Madison, WI, USA). Sample cDNA was pipetted onto the array PCR plate with Taqman Fast Advanced Mastermix (Applied Biosystems, Foster City, CA, USA) to detect genes involved in the bone repair process (Taqman Array Fast 96 well plate, Applied Biosystems). Real-time PCR was performed on Step One Plus real-time PCR detection system (Applied Biosystems) under the following conditions: 50 °C (2 min), 95 °C (10 min), 40 cycles of 95 °C (15 s), and 60 °C (1 min), followed by a standard denaturation curve. The relative gene expression was calculated by referencing the mitochondrial ribosomal protein expression and normalized by the gene expression of calvaria fragments undergoing repair during different experimental periods (ΔΔCT method). The assay was performed in quadruplicate, and the genes evaluated were Vascular Endothelial Growth Factor (VEGF), RUNX Family Transcription Factor 2 (RUNX2), and Integrin Binding Sialoprotein (IBSP), with Beta-actin (β2-Microglobulin, B2M) as constitutive.

### 2.6. Statistical Analysis

The statistical analysis was performed using GraphPad Prism software (GraphPad Software, Inc.; San Diego, CA, USA; 2017). Normal distribution analysis was performed using the Shapiro–Wilk test to distinguish parametric and nonparametric data. One-way ANOVA and Tukey’s post-test were used to analyze the micro-CT, histometric, and real-time PCR data, adopting a significance level of 95% and a study power of 80% for all tests.

## 3. Results

### 3.1. Histometric Analysis

**Figure 2:**
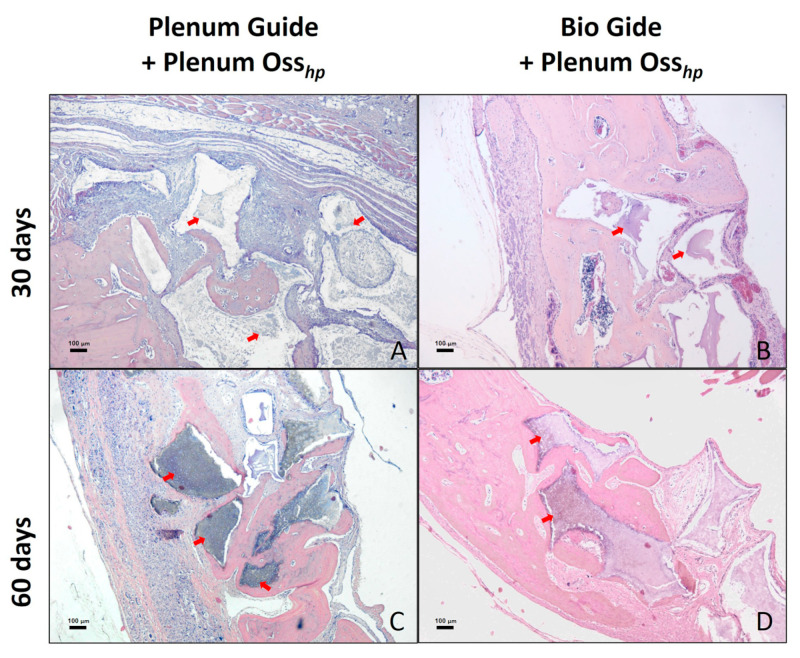
The histological aspect of the surgical defect area in calvaria and the adjacent regions. Photomicrograph showing histological characteristics of the connective tissue, bone tissue, and remaining biomaterial at 30 days postoperatively in groups PG (**A**) and BG (**B**). Observe a larger area of bone formation at 60 days postoperatively in the PG (**C**) and BG (**D**) groups. The arrows indicate the presence of remaining biomaterial. Staining: HE. Original magnification: 50x. Scale bars: 100 μm.

Newly formed bone tissue, connective tissue, and the remaining synthetic bone graft (Figure 2) were quantitatively analyzed in volumetric percentages of the evaluated samples, as shown in Table 2.

At 15 and 30 days, there was a higher percentage of connective tissue in the PG group compared to the BG group (*p* < 0.05). At 60 days, there were no statistically significant differences between the groups, suggesting an advance of the bone repair process in the PG group over time. 

Regarding the percentage of bone tissue, no statistical differences were found in the periods analyzed. However, a greater amount of bone tissue formed at 30 days was observed in the BG group when compared to the PG group. However, these values are equalized between the groups in the last period of analysis, at 60 days. 

The PG group showed a higher percentage of material remnants than the BG group at 15 days (1.07 ± 0.4 versus 0.51 ± 0.08). However, at 30 and 60 days, no statistically significant differences were observed between these periods (p>0.05).

### 3.2. Immunolabeling Analysis

**Figure 3:**
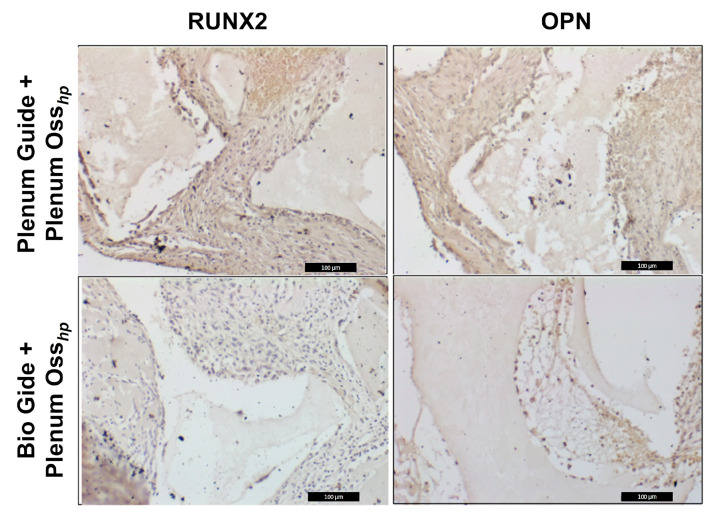
Immunolabeling using RUNX2 and OPN in the period of 7 days.

In the PG group, at seven days, a moderate immunostaining (2) for RUNX2 was found, especially in the area near the biomaterial, as well as a moderate immunostaining (2) for OPN, both in cells of the osteoblastic lineage and in the extracellular matrix. For the BG group, in the same period, we observed a mild (1) immunostaining of RUNX2 in cells of the osteoblastic lineage and a mild (1) immunostaining for OPN, especially in cells of the osteoblastic lineage (Figure 3).

### 3.3. Real-Time PCR Analysis

**Figure 4:**
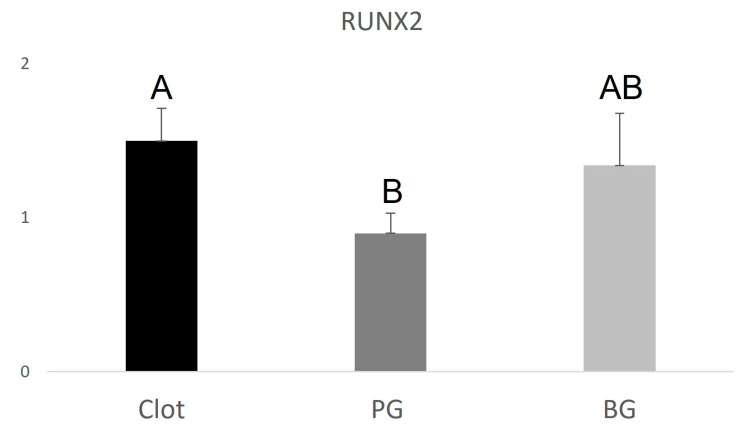
Graphical representation of the relative RUNX2 gene expression of the PG and BG groups when compared to clot. Distinct letters indicate statistical differences.

**Figure 5:**
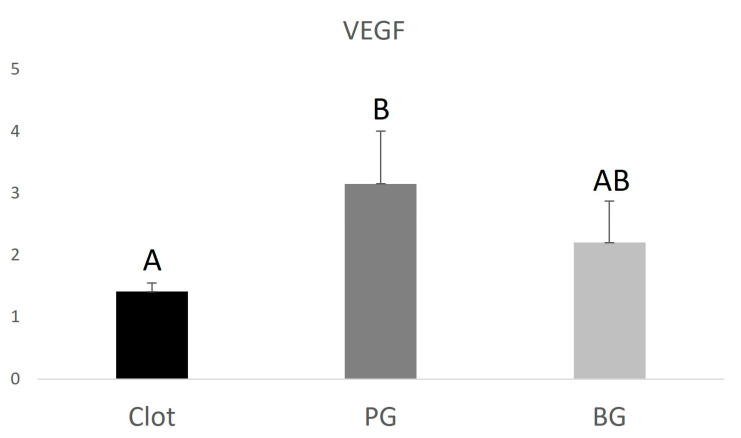
Graphical representation of the relative VEGF gene expression of the PG and BG groups when compared to clot. Distinct letters indicate statistical differences.

**Figure 6:**
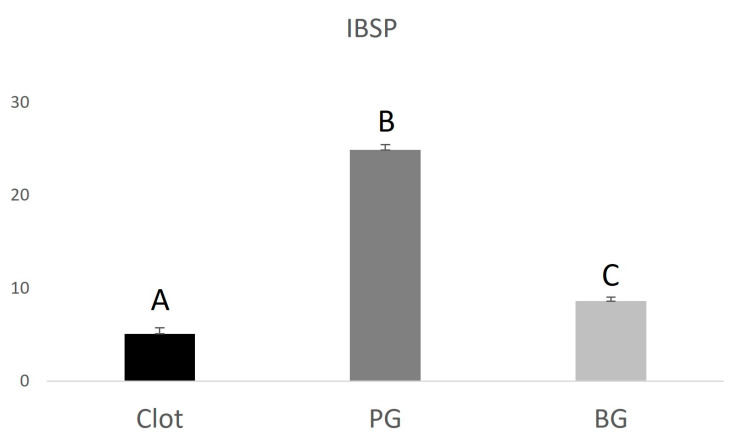
Graphical representation of the relative IBSP gene expression of the PG and BG groups when compared to clot. Distinct letters indicate statistical differences.

The PG group (0.9 ± 0.130.22) showed lower RUNX2 expression when compared to the BG group (1.34 ± 0.34), higher VEGF expression (3.15 ± 0.85) compared to the BG group (2.2 ± 0.67), and higher IBSP expression (24.9 ± 0.59) compared to the BG group (8.6 ± 0.43). The data are represented in Figure 4, Figure 5 and Figure 6 below.

### 3.4. Micro-CT Analysis

The threshold for mineralized tissue: for the parameters BV/TV, TbN, and TbTh, there were no statistically significant differences between the groups evaluated (*p* > 0.05). For Tb.Sp, no significant differences were observed between the PG groups, 0.36 (0.3–0.48), and the BG group, 0.37 (0.33–0.6). For the ConnDn parameter, no significant differences were observed between the PG groups, 70.28 (1.86–134.84), and the BG group, 26.42 (0.58–57.16).

**Figure 7:**
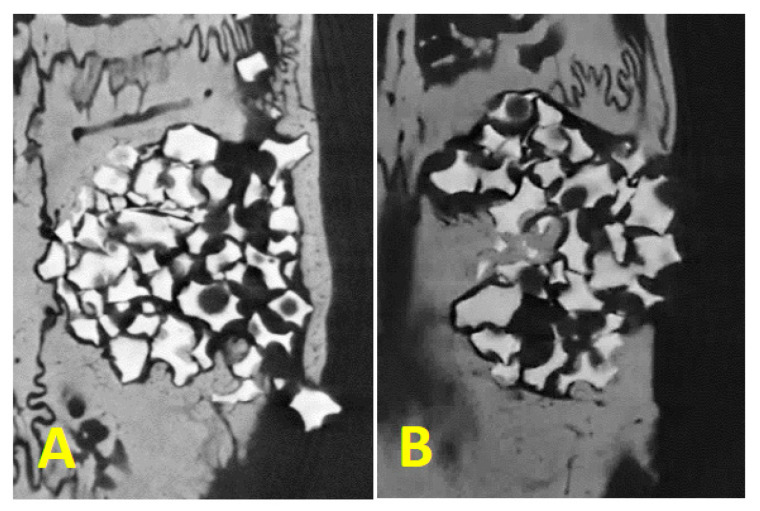
Mineralized tissue inside the critical defect crossing the biomaterial granules being (**A**): Plenum^®^ Guide + Plenum^®^ Oss*_hp_* and (**B**): Bio Gide^®^ + Plenum^®^ Oss*_hp_*.

The threshold for remaining regenerative material: the same parameters (BV/TV, TbN, TbTh, TbSp, and ConnDN) were evaluated, with adjustment for remaining biomaterial Plenum^®^ Oss*_hp_*, and no statistical differences were found for any of the parameters between the groups (Figure 7).

## 4. Discussion

The present study showed that Plenum^®^ Guide could be used to regenerate bone tissue in critical defects of rat calvaria. A higher amount of RUNX2 and OPN immunolabeling was obtained, denoting better cellular responses in the group in which Plenum^®^ Guide was used for GBR compared to the collagen membrane. Furthermore, the PG group showed a higher relative gene expression of VEGF. This vascular endothelial growth factor plays an essential role in blood vessel proliferation and migration of osteoprogenitor cells, contributing directly to the bone repair process. As well as for newly formed bone tissue in the defect area, the PG and BG groups obtained similar results.

Immunohistochemical analysis showed a higher expression of RUNX2 and OPN in the PG group. RUNX2 is a major transcription factor that regulates osteoblast activity and mesenchymal cell differentiation, playing a critical role in bone formation. Consistent with this finding, RUNX2-deficient mice lack mature osteoblasts, and the bone formation process is incomplete [29]. On the other hand, OPN, secreted by osteoblasts, is one of the significant non-collagenous proteins in the bone matrix. In addition, OPN is involved in bone mineralization [30]. These findings suggest a higher quality of bone tissue found in the PG group than in the BG group, where the same proteins were observed discretely. This characteristic influences the maintenance of long-term bone levels, which is essential when considering using such biomaterials for oral rehabilitation with osseointegrated implants.

As for relative gene expression, the BG group obtained higher expression of RUNX2 when compared to the PG group, indicating more signaling for the production of this factor during the bone repair process. Collagen-based membranes are widely used in sinus augmentation, grafting techniques for gaining alveolar ridge thickness and height, and other techniques that comprise guided bone regeneration, with good results [31,32,33]. Collagen is the extracellular matrix’s main component (ECM) and is involved in critical cellular processes, including adhesion, proliferation, migration, and cell differentiation. [34] Collagen membranes have been widely used in guided bone regeneration (GBR), a technique often used to augment alveolar ridge volume deficiencies to ensure successful implant placement. However, heterogeneous biomaterials tend to provoke higher inflammatory levels and unwanted immunogenic responses, often making their use unfeasible [35,36,37].

VEGF and IBSP gene expression were shown to be higher in the PG group. Vascular endothelial growth factor (VEGF) is an angiogenic cytokine with direct action on endothelial cells, which plays a crucial role in regulating osteogenesis/angiogenesis interaction. Osteoblast-derived VEGF is essential for bone development and maintenance of bone homeostasis. Studies have shown that manipulating VEGF levels can affect bone repair because this factor is directly related to blood vessel proliferation. With the invasion of blood vessels, osteochondrocyte mesenchymal progenitor cells migrate to the repair site, where they proliferate and differentiate into either osteoblasts or chondrocytes, inducing the newly formed bone to be gradually remodeled into lamellar bone [38]. IBSP is a glycoprotein that plays a key role during the alveolar repair process due to its osteogenic property, acting in the differentiation of bone marrow precursor cells into osteoblasts [39].

A greater amount of remaining connective tissue was found in the PG group at 15 and 30 days postoperatively. This period is considered initial for the bone repair process, being consolidated after 60 days, when the amount of connective tissue in both groups was equal. This finding may be directly related to the presence of biomaterial found in the PG group at 15 days, suggesting a more prolonged action of the PDO membrane when compared to the collagen membrane. Studies show that a longer absorption time favors bone regeneration.

Despite the more significant amount of connective tissue, there was no statistical difference regarding bone formation between the PG and BG groups. Both groups achieved promising results in the 60-day evaluation period. The performance of both membranes was similar when analyzed by computed microtomography, with no statistical differences between the PG and BG groups. Considering the final amount of bone formation, it was noted that both the polydioxanone membrane (Plenum^®^ Guide) and the collagen membrane (Bio Gide^®^) had similar behaviors, acting as barriers for non-osteogenic cells and favoring an adequate bone regeneration process. It is essential to point out that collagen or chitosan membranes have a strong immunogenic response associated with most natural polymers, complexities related to their purification, and the possibility of disease transmission [16]. Other disadvantages associated with using collagen membranes for GTR or GBR are the loss of space-maintaining ability under wet conditions [38,40], lower mechanical strength, and faster degradation [37]. These limitations, such as poor mechanical properties and rapid degradation, are associated with a decreased period of the function of such materials, resulting in increased susceptibility to infection and undesired tissue regeneration [35,41].

Regarding the amount of bone graft remaining, an increase was verified in the control group, from the initial period to the 60-day period, which can be explained by the lack of weighing of the grafted material on the defects, only one instrument measured with visual inspection was used to fill the defects, which resulted in groups with more particles and groups with fewer particles of the biomaterial, but this factor did not alter the analysis of the desired results.

The excellent performance of collagen membranes for guided bone regeneration is irrefutable; however, it is of fundamental importance to develop new synthetic biomaterials to expand the possibilities in the field of guided bone regeneration since the use of biomaterials of animal origin is unfeasible. Plenum^®^ Guide presents all the advantages of a synthetic absorbable membrane, and the results demonstrated a performance similar to collagen membranes for GBR regarding to neoformed bone micro-architecture.

## 5. Conclusions

In general, both membranes promote predictable results for bone regeneration, with well-structured neoformed bone tissue, as evidenced by the micro-architecture from the micro-CT and histological analysis. Hence, PDO membranes in this experimental study presented higher expression to immunolabeling than collagen membranes, a well-known key factor in early bone tissue neoformation, demonstrating excellent performance and safety as a medical device. Furthermore, synthetic membranes can be a viable option for absorbable synthetic material for GBR applications, primarily owing to ethnic and religious issues. 

## Figures and Tables

**Table 1:** Taqman probes for real-time PCR.

Gene	Gene Name	Identification
B2M	B2M	Rn00560865_m1
RUNX-2	RUNX2	Rn01512298_m1
VEGF	VEGFA	Rn01511602_m1
IBSP	IBSP	Rn00561414_m1

**Table 2:** Histometric analysis of the groups in the different periods evaluated.

	Period (days)	Plenum^®^ Guide + Plenum^®^ Oss*hp*	Bio Gide^®^ + Plenum^®^ Oss*hp*
**Connective Tissue**	15	1.63 (0.36)a	0.86 (0.17)b
	30	1.32 (0.53)ab	0.81 (0.2)b
	60	0.83 (0.45)b	0.70 (0.34)b
**Newly formed bone tissue**	15	0.38 (0.26)a	0.21 (0.11)a
	30	0.40 (0.23)a	0.68 (0.21)a
	60	0.60 (0.24)a	0.65 (0.51)a
**Bone graft remaining**	15	1.07 (0.4)a	0.51 (0.08)b
	30	0.87 (0.36)a	0.78 (0.26)a
	60	0.84 (0.31)a	0.91 (0.33)a

Note: Different lowercase letters (a and b) indicate the statistical difference between the groups (*p* > 0.05).

## Data Availability

The data presented in this study are available on request from the corresponding author.
